# Supplementary material for: Glyoxal Fixation Is Optimal for Immunostaining of Brain Vessels, Pericytes and Blood-Brain Barrier Proteins
Source: Int J Mol Sci. 2022 Jul 14;23(14):7776. doi: 10.3390/ijms23147776 (PMC9317650; doi:10.3390/ijms23147776)
Supplement: Supplementary file 1 [file ijms-23-07776-s001.zip › ijms-1758097-supplementary.pdf]

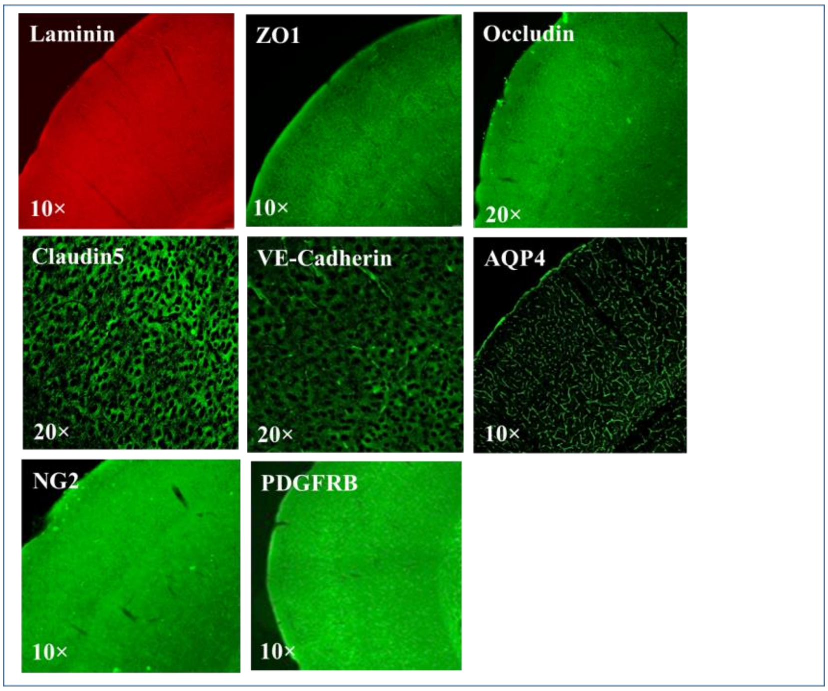


**Supplementary Figure S1.** Immunohistochemistry staining of vascular proteins laminin, Claudin5, VE-Cadherin,  Occludin, ZO-1, AQP4 and pericyte marker NG2 in PFA fixed brain sections. Except AQP4 and VE-Cadherin all other staining didn't show any positive signals in immunostaining of PFA fixed brain.
